# Would Financial Development Help China Achieve Carbon Peak Emissions?

**DOI:** 10.3390/ijerph191912850

**Published:** 2022-10-07

**Authors:** Ping Ji, Weidong Huo, Lan Bo, Weiwei Zhang, Xiaoxian Chen

**Affiliations:** 1 International Business School, Southwestern University of Finance and Economics, Chengdu 611130, China; 2 School of Finance and Trade, Liaoning University, Shenyang 110136, China; 3 Sunwah International Business School, Liaoning University, Shenyang 110136, China

**Keywords:** financial development, carbon intensity, technological innovation, industrial upgrading, spatial econometric model

## Abstract

China has committed to reaching carbon peak before 2030. To realize the carbon peak goal, financial development plays an essential role in developing a green economy. Based on the panel data of 30 provinces in China from 2006 to 2019, this paper explores the impact of financial development on carbon intensity both theoretically and empirically. A financial development index system is constructed and computed using the entropy method. A spatial lag panel data model is employed to empirically test the interaction effect of financial development on carbon intensity. Moreover, the mediating effects of industrial upgrading and technological innovation are further investigated. The results show that: first, carbon intensity generates strong spatial spillover effects between provinces in China. Second, financial development significantly reduces carbon intensity, and is most pronounced in central China, followed by western and eastern China. Third, industrial upgrading and technological innovation are important channels to assist financial development in cutting down carbon intensity, and both produce positive spatial spillover effects. These findings suggest that inter-regional cooperation and coordination on financial development, industrial upgrading, and technological innovation are conducive to achieving low-carbon development targets. This research not only has practical significance to China, but also provides global reference value to other countries.

## 1. Introduction

The Chinese economy has made rapid progress since the reform and open-up in 1978. However, the extensive development mode of high consumption, high emissions, and low efficiency has caused the relentless rise of carbon dioxide and other greenhouse gas emissions, which has brought great damages to the ecological environment [1,2]. At present, China is facing severe downward economic pressure, and the new development philosophy featuring “innovative, coordinated, green, open and shared development” has been put forward to accommodate to the “economic new normal” and “supply-side structural reform”, which indicate that China’s economy will change to the direction of intensive and sustainable development [3,4]. At both the 75th and 76th Session of the UN General Assembly in 2020 and 2021, President Xi pledged that China will hit emissions peak before 2030 and achieve carbon neutrality before 2060. In the 14th Five-Year Plan, China will resolutely give priority to ecological development, promote low-carbon green growth, and continuously improve the ecological civilization system, with the aim of contributing to the global response to climate change. The 2021 Chinese Government Work Report also clearly states “achieving carbon emissions peak and carbon neutrality” is one of the key tasks for governments. To fulfill the “Double Carbon” goal, China must cut down its carbon emissions and develop a low-carbon economy. The financial market plays an essential role in helping ensure carbon reduction. Therefore, this paper investigates the influence of financial development on emission reductions and explores the impact mechanism of the relationship both theoretically and empirically. The findings in this article are enlightening to both China and other countries that are trying to cut down carbon emissions.

The earliest literature on carbon emission dates from the academic research on the association between environment and economy, i.e., the theoretical and empirical analysis of environmental Kuznets curve (EKC), which verifies whether there is an “inverted U-shaped” relationship between environmental pollution and economic growth. Some studies confirmed the validness of the EKC hypothesis [5,6], but others hold a different view [7,8]. Then, in the late 1980s, research on carbon emissions began. Japanese scholar Kaya [9] quantified the relationship between economy, population, energy factors, and carbon emissions. Hereafter, a large number of time series analyses, focusing on the determinants of carbon dioxide emissions, demonstrated that economic growth, trade openness, energy consumption, industrial structure, and government financial expenditure are all important factors affecting carbon emissions [10,11,12].

Extant studies drew different conclusions about the impact of financial development on carbon emissions. First, financial development can reduce carbon emissions and assist the development of a low-carbon economy. Since regions with a higher level of financial development are capable of attracting foreign direct investment (FDI) and enjoying a series of induced effects brought by FDI, accordingly, they can cut down emissions and improve environmental quality [13,14]. Second, there is an inverted U-shaped relationship between financial development and carbon emissions [15,16]. This means that, while financial development boosts economic growth, it also induces more energy consumption and emissions [17,18]. Nevertheless, after a certain level, the advancement of financial development propels the progress of technology, which consequently lowers carbon emissions [19,20]. Third, there is a threshold effect between financial development and carbon emissions. In other words, finance only stimulates emission reduction in the later stage of economic development. In an emerging market with low financial development, finance is not virtually effective against carbon emissions from industries, therefore the influence of financial development on carbon reduction is not apparent [21,22].

Likewise, studies specifically focusing on China showed inconsistent outcomes. Some have shown that China’s financial development can improve environmental quality [23,24], but others believe that financial development causes the increase in household energy consumption, thereby leading to lower environmental quality [25]. In addition, Yin, et al. [26] believe that financial development in China creates more environmental benefits in financially developed areas, but not in financially backward areas.

In terms of the measurement of financial development, the vast majority of current studies adopt a single indicator method, for instance, bank’s assets/GDP [27], M2/GDP [28], or banks’ credit/GDP [29,30], etc. Most of these indicators only reflect the development of the banking sector in the financial market. With continuous development, banking, insurance, and securities all become the key sectors of the financial industry, which has gradually become the “blood” of the modern economy.

The existing literature provides us with the fundamental theoretical support and empirical evidence in the related research, but there are still some areas for improvement: first, the selection of a single financial development indicator does not fully capture the characteristics of financial development, which may affect the robustness of empirical results; second, most research methods do not consider the impact of the geographical environment on carbon emissions; third, the impact mechanism between financial development and carbon emissions has rarely been explained and tested.

Given the limitations in the extant literature, this paper may have the following contributions. First, rather than using a single factor, we consider the development of the banking, insurance, and securities sectors together and adopt the entropy method to weigh and calculate the financial development index from multiple indicators. Second, in view of the influence of carbon dioxide on the neighboring areas, the spatial interactive effects of financial development on the carbon intensity are analyzed, which enriches the research on the relationship between financial development and environmental pollution. Third, considering the endogeneity of the IPAT framework [31], the mediating effect of industrial upgrading and technological innovation on the relationship between financial development and carbon intensity is investigated, which provides more insights into the role of the financial market.

The rest of this paper is organized as follows: Section 2 is the theoretical analysis on the impact mechanism of financial development on carbon emissions. Section 3 explains the construction of the financial development index system, the measurement of carbon intensity, the definition of mediating and other control variables, as well as data sources. Section 4 illustrates the methodologies, including the setting of spatial weight matrix and spatial lag model. Section 5 analyses the empirical results of spatial autocorrelation test, spatial lag effect estimation, and mediating effect. The last part comprises the conclusions and policy implications.

## 2. Theoretical Analysis

### 2.1. Impact Mechanism via Industrial Upgrading

Financial development could influence carbon emissions through industrial upgrading. Firstly, as an important part of the tertiary industry, the finance sector itself is a low-carbon industry and its development could crowd out or substitute other industries. The role of the finance sector naturally makes ways for the upgrade and expansion of the tertiary industry and thereby reduces carbon emissions generated by other industries [32,33]. Secondly, the finance sector can allocate financial capital to different industries both directly and indirectly. The direct way refers to the direct financing of enterprises through the capital market, for example, green and low carbon enterprises can issue green bonds for green technological innovation and development, or transfer equity shares to realize mergers and acquisitions and optimize capital allocation. In this manner, finance development can directly improve the industrial structure and help achieve low-carbon development [34,35]. The indirect way means that commercial banks and other financial institutions can guide the flow of financial capital by providing concessionary loans to low-carbon industries. They either refuse or require higher interest rates to high pollution, high energy consumption, and high emission industries. In this way, the environment contamination behavior is punished by financial institutions [36,37]. Therefore, the finance sector can assist in the transformation and upgrading of industries, along with the simultaneous reduction of emissions by just picking the fittest enterprises. In addition, policy-based finance is a powerful means to push for the advancement of industrial structure and accomplish low-carbon development. The Chinese government has formulated a series of financial policies to support industrial development. Financial institutions can offer policy-based loans, credit insurance, interest subsidies, and other special financial services to qualified enterprises and individuals [38,39]. For instance, China’s Inclusive Finance policy was implemented to aid all social strata, including vulnerable and weak groups, and helps tackle the financing difficulties of small and medium-sized enterprises, which not just diversifies the capital market structure but benefits more high-tech companies. Financial institutions also supply loans to projects associated with rural area development and farmers’ welfare at a preferential interest rate. By doing so, financial institutions promote the modernization, industrialization, and low carbonization of the agriculture sector. These policies essentially give impetus to the structure optimization of traditional industries and lead to the development and growth of the green economy.

### 2.2. Impact Mechanism via Technological Innovation

Financial development can also influence carbon emissions through technological innovation. Firstly, financial institutions could redistribute the social resources to green and low-carbon industries and boost technological innovation and creativity by the invisible hand of the financial market [40,41,42]. Financial resources can be converted from savings to investment through the government and financial institutions in the monetary market, which are accordingly being invested in environmental protection technology [25,43]. Meanwhile, the progress of low-carbon technology and the transformation of science research into real productivity all require tremendous social resources, which they cannot obtain without the funding support of financial markets. Secondly, financial institutions can accelerate the evolution of low-carbon technologies by facilitating the accumulation of human capital. As the carrier of technological progress, human capital is the main expression of endogenous technological progress in the neoclassical growth theory [44,45]. Education investment is the key to the formation of human capital. Due to its high cost and long payback period, it is difficult to obtain the necessary returns with private education investment. Therefore, large financial entities such as banks, insurance, and securities institutions are needed to provide essential financial support for human capital development in universities and research institutes, with the aim to cultivate high-tech talents and hence promote the progress of low-carbon technologies. Furthermore, finance development motivates the progress of low-carbon technologies by influencing technology spillovers. Comparable with FDI technology, financial development promotes growth on technological progress and industrial innovation across different regions [46,47]. Financial investment in new machines or hiring workers with new skills not merely improves productivity and reduces costs in the local area, but also narrows the technological gap between regions through the flow of technical staff. Thereby the overall technological innovation stimulated by financial development can contribute to carbon reduction.

**Figure 1:**
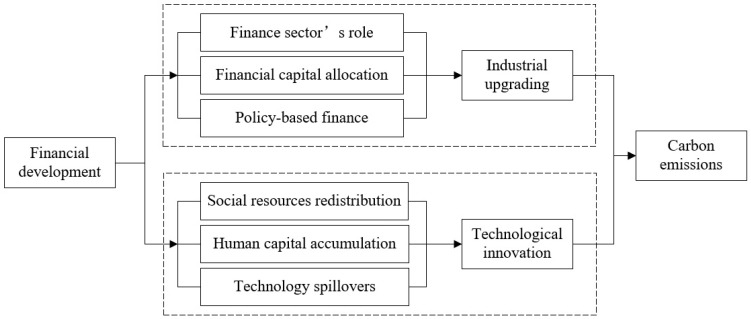
Impact mechanism analysis.

The impact mechanism analysis of industrial upgrading and technological innovation on financial development and carbon emissions is shown in Figure 1.

### 2.3. Extended IPAT Framework Analysis

The IPAT framework proposed by Dietz and Rosa [31] decomposes the driving forces of the environment changes into three key features of human dimensions: population, affluence and technology, which is depicted as the STIRPAT (stochastic impacts by regression on population, affluence, and technology) model and illustrated by Equation (1):

(1) $$I_{it}=aP_{it}^{b}A_{it}^{c}T_{it}^{d}e_{it}$$

where *I*, *P*, *A*, and *T* stand for environmental impact, population, affluence, and aggregated technology-related activities, respectively.

As the affluence *A* in the STIRPAT model represents the aggregated economic activities in the society, it can be substituted by financial market activities to accommodate our analysis. *T* can also be interpreted as industrial upgrading or technological innovation and all other factors are grouped into the residual term ***e***. Considering the endogeneity of industrial upgrading or technological innovation, which are motivated by financial development, we extend the IPAT framework by importing the mediating effect of technology *M* as Equations (2) and (3):

(2) $$M_{it}=\alpha P_{it}^{\beta }A_{it}^{\gamma }\varepsilon_{it}$$

(3) $$I_{it}=aP_{it}^{b}A_{it}^{c}M_{it}^{d}e_{it}$$

The remaining empirical analysis will be derived from the extended IPAT framework to investigate the impact of financial development on carbon reduction and the mediating effect of industrial upgrading and technological innovation.

## 3. Indicators, Variables and Data

### 3.1. Financial Development Indicators

The World Bank [48] defines financial development as the process of overcoming costs incurred in the financial system when financial instruments, markets, and intermediaries providing the key functions of the financial sector in the economy. Therefore, a single indicator cannot fully reflect the financial sector development. Different from Adu, et al. [49]’s principal component analysis method, which focuses on the banks’ credit to private-sector and broad-narrow money, we consider factors to measure financial development from various aspects such as the development of financial institutions, the environment of the finance sector, and the output of the financial market.

A comprehensive index system of financial development is constructed by 12 indicators. The development of financial institutions is proxied by deposits and loans of the banking sector, income and expenditure of the insurance sector, proceeds of bonds and shares in the securities market, and the number of financial institutions. The environment of the financial development is proxied by the investment of fixed assets, the number of financial practitioners, and their average salaries. Finally, the output of the financial market is measured by GDP of finance sector, etc. As the indicators are from different financial sub-sectors of different provinces at different times, the entropy weight method is used to derive the index of financial development. The greater the dispersion degree of data, the more the information content, and the higher weight will be given to the indicator [50]. The specific steps are as follows:

(1) Data standardization

Assume the dataset includes *m* indicators of *t* years from *n* provinces. $X_{\eta ij}$ represents indicator *j* of province *i* in year *η*, which is standardized by its mean as:

(4) $$X_{\eta ij}^{\prime }=X_{\eta ij}/\bar{X_{\eta ij}}$$

(2) Information entropy valuation

$Y_{\eta ij}$, the probability parameter for indicator $X_{\eta ij}$, is calculated as the proportion of $X_{\eta ij}^{\prime }$ to this indicator in all provinces for all years; $e_{j}$ denotes the information entropy of indicator *j*.

(5) $$\{\begin{array}{c} Y_{\eta ij}=\frac{X_{\eta ij}^{\prime }}{\sum_{\eta =1}^{t}\sum_{i=1}^{n}X_{\eta ij}^{\prime }}\\ e_{j}=-\kappa \overset{t}{\underset{\eta =1}{\sum }}\overset{n}{\underset{i=1}{\sum }}Y_{\eta ij}\ln Y_{\eta ij}\\ \kappa =\frac{1}{\ln\;tn}>0 \end{array}$$

(3) Entropy weight determination

$w_{j}$, the weight of indicator *j*, is determined as follows:

(6) $$w_{j}=\frac{1-e_{j}}{\sum_{j=1}^{m}1-e_{j}}$$

(4) Index calculation

With the entropy weight, the financial development index of province *i* in year *η* is as follows:

(7) $$U_{\eta i}=\overset{m}{\underset{j=1}{\sum }}w_{j}X_{\eta ij}^{\prime }$$

Table 1 summarizes the definitions, the entropy values, and the weights of all 12 indicators.

### 3.2. Variable Selection

#### 3.2.1. Dependent Variable

**Figure 2:**
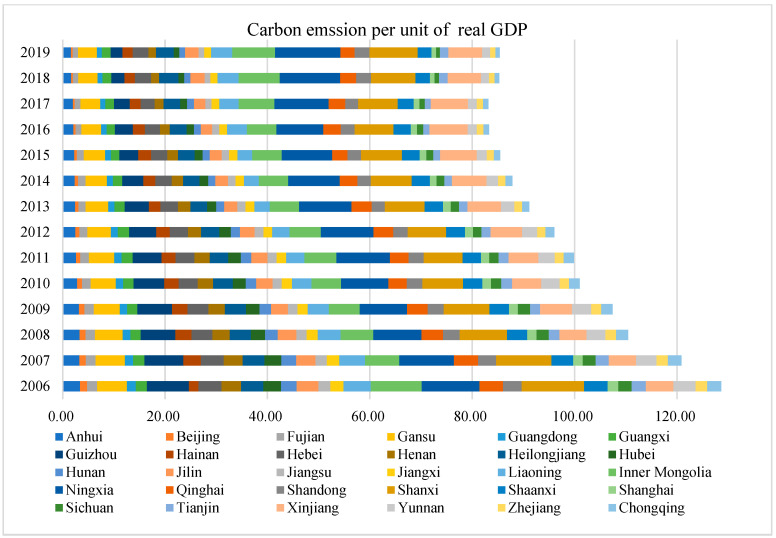
Provincial carbon intensity from 2006 to 2019.

Carbon intensity (CI) is the dependent variable to be examined in this paper. Following the 2006 IPCC guidelines, nine kinds of energy—coal, coke, crude oil, gasoline, kerosene, diesel, natural gas, fuel oil, and electricity—were taken as consumption by end-users in each province, and converted to a common energy unit and multiplied by emission factor to work out the carbon dioxide emissions. We define the carbon intensity as the provincial carbon emissions per unit of real GDP. Figure 2 plots the emission intensity of 30 provinces (30 provinces virtually include provinces, autonomous regions, and municipalities directly under the Central Government. For brevity, “30 provinces” is the term used in the rest of the article) in China from 2006 to 2019. It shows the overall carbon intensity in provinces of China are declining over the years.

**Figure 3:**
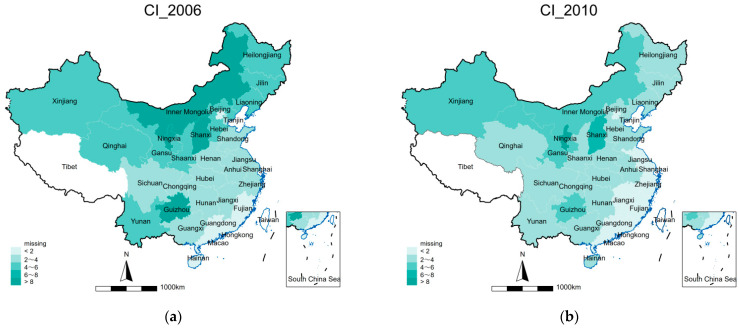

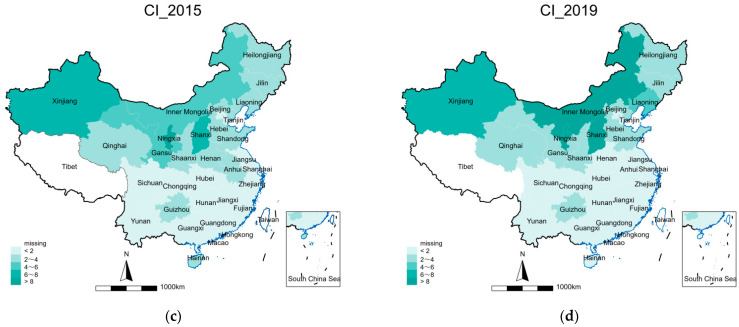
(**a**–**d**) Geographical distribution of provincial carbon intensity in 2006, 2010, 2015, and 2019.

The geographical distribution of provincial carbon intensity in 2006, 2010, 2015, and 2019 are depicted in Figure 3. The darker the color, the greater the carbon intensity. We can expect more pollution and worse environmental quality in darker regions. As can be seen, the regions with high carbon intensity are clustered in northern China, such as Xinjiang, Inner Mongolia, and Shanxi. The intensity in southern China is relatively smaller, indicating that emission magnitude in China has certain characteristics of aggregation. In addition, while that in southern China was mostly decreasing from 2006 to 2019, carbon intensity in northern China generally intensified over the same period.

#### 3.2.2. Explanatory Variables and Mediating Variables

Financial development (FD): Financial development index determined by the entropy weight method is used as the key explanatory variable in this paper to explore its spatial effect on carbon intensity.

Industrial upgrading (Stru). Following Moore’s method [51], the change index of Moore structure is used to measure the degree of industrial structure upgrading of 30 provinces in China, as shown in Equation (8):

(8) $$\{\begin{array}{c} Moore_{t}=\frac{\sum_{i=1}^{3}\left(w_{i,0}\times w_{i,t}\right)}{{\left(\sum_{i=1}^{3}w_{i,0}{}^{2}\times \sum_{i=1}^{3}w_{i,t}{}^{2}\right)}^{\frac{1}{2}}}\\ Stru=arccos\left(Moore_{t}\right) \end{array}$$

where *i* (*i* = 1, 2, 3) represents the primary, secondary, and tertiary industry, respectively; 0 is the base year, *t* is the comparative year (*t* = 2006–2019); $w_{i,t}$ and $w_{i,0}$ represent the proportion of the *i*th industry in GDP by different provinces in year *t* and the base year, respectively; $Moore_{t}$ designates the cosine of the vector angle formed by $w_{i,t}$ and $w_{i,0}$; *Stru* represents the index of industrial upgrading. The larger the index value, the higher level of the industrial upgrading is, and vice versa.

Technological innovation (Tech): To measure the technological innovation capability of regions, the entropy weight method is again applied to construct a technological innovation index by using the following indicators: the number of scientists and engineers, the number of licensed patents, the amount of R&D expenditure, and the turnover of technological market. The greater the index, the more innovative the province is.

#### 3.2.3. Control Variables

Degree of openness (Open): With the rapid development of the economy and increasing opening-up to other economies, China’s imports and exports have sustained steady growth over the decades. Regional imports and exports directly reflect the level of the local economic ties with other countries, therefore, the percentage of total provincial imports and exports over GDP represents the degree of openness.

Foreign investment contribution (FDI): The pollution haven hypothesis reflects the impact of foreign direct investment (FDI) on environmental pollution. The general view of the hypothesis states that the inflow of FDI will aggravate environmental pollution in host countries [52]. However, Akar [53] argues there is no clear evidence to prove this hypothesis. Zhou, et al. [54] also show that FDI can assist regional economic development, provide financial support, accelerate industrial structural upgrading, and benefit the environment. To test the impact of FDI on carbon intensity in China, the percentage of provincial FDI over GDP is used to measure the contribution of foreign investment.

Government intervention (Gov): Moderate government intervention can help to make up for market regulation defects by providing policy support to sustainable industries, which is conducive to the reduction of regional carbon emissions [55]. Nevertheless, excessive or lack of government intervention without correcting the problems caused by market failure can lead to a certain degree of environmental pollution [56]. Therefore, the percentage of provincial government fiscal expenditure over GDP is used to proxy the degree of government intervention in the economy.

Environmental regulation (Reg): It is generally believed that the stronger the environmental regulation, the lower the carbon emissions will be [14]. However, will there be a reverse relationship of “the more regulation, the more pollution”? Here, Levinson’s approach is adopted to measure the stringency of environmental regulations of each province of China [57]. The specification of Reg is defined as follows:

(9) $$Reg_{it}=Q_{it}/Y_{it}$$

where $Q_{it}$ and $Y_{it}$ represent the investment in industrial pollution control and the industrial output of province *i* in year *t*, respectively. Due to the structural differences between provinces, Equation (9) is modified to reflect the industrial structural differences, as shown below:

(10) $$\{\begin{array}{c} S_{it}=Y_{it}/GDP_{it}\\ Reg_{it}^{*}=Reg_{it}/S_{it}\times 100 \end{array}$$

where $S_{it}$ is the industrial structure and $Reg_{it}^{*}$ is the modified index of government regulation.

### 3.3. Data Sources and Descriptive Statistics

The panel data of 30 provinces in China (excluding Hong Kong, Macao, Taiwan, and Tibet) was collected from 2006 to 2019. The consumption of different energy used to calculate carbon intensity are from China Energy Statistical Yearbook (2007–2020). Data used to construct the indexes system of financial development and technological innovation, as well as the control variables, all stem from China Statistical Yearbook (2007–2020). Most missing data could be found in the Statistical Bulletin of National Economic and Social Development of Provinces (2006–2019). If it could not be obtained anywhere, a scientific and technical approach was adopted to replace the missing value.

Logarithms of all variables with different units and magnitudes were taken to correct heteroscedasticity. To eliminate the impact of price fluctuations, 2006 was chosen as the base year. Data in US dollars, such as imports, exports and FDI, are converted into RMB by the end-of-period exchange rates. Statistical analysis is done by STATA 16 software, and the descriptive statistics of 420 observations for each variable are given in Table 2.

**Figure 4:**
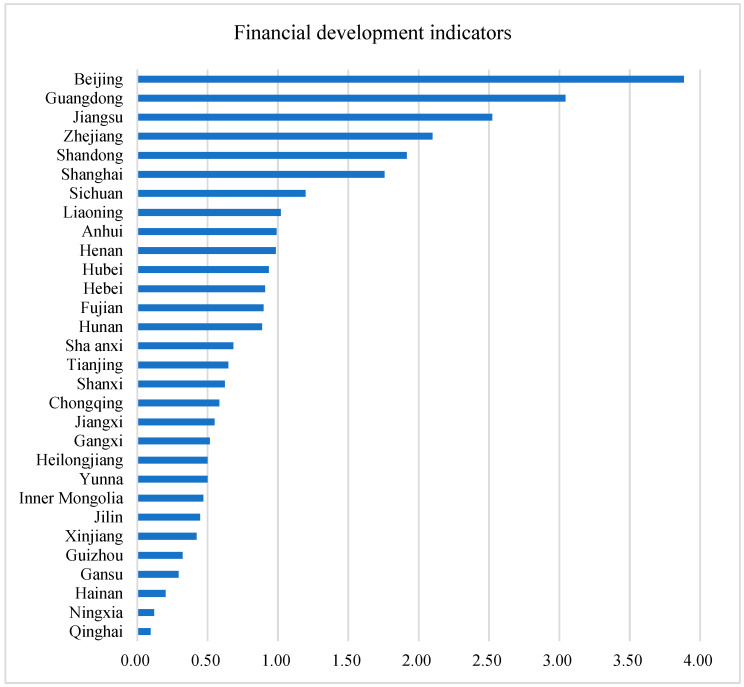
Provincial average financial development indexes from 2006 to 2019.

Figure 4 describes the provincial average financial development indexes from 2006 to 2019. In terms of financial development level, the top three provinces are Beijing, Guangdong, and Jiangsu, and the last three are Qinghai, Ningxia, and Hainan. Nationally, the average financial development index increased from 0.336 in 2006 to 1.550 in 2019, with an average annual increase rate of 25.82%.

## 4. Methodologies

With the development of financial technology, regional financial investment behavior has an apparent “spatial effect”, which may break the distance constraints between countries, alleviate the problem of information asymmetry, and realize better spatial links of financial resources. Therefore, spatial factors can be incorporated into the classical regression model that is modified and improved through spatial econometric estimation. Compared to other empirical models, a spatial econometric model that integrates regional, location, and spatially related factors has its advantages in investigating financial problems.

### 4.1. Geographical Weight Matrix

Geographical weight matrix *W*^1^ is used to measure the spatial relationships between 30 provinces in China, as defined below:

(11) $$W^{1}=\left[\begin{array}{ccc} w_{1,1} & \cdots & w_{1,30}\\ \vdots & \ddots & \vdots \\ w_{30,1} & \cdots & w_{30,30} \end{array}\right]$$

where $w_{i,j}$ represents the weight matrix of geographical distance between province *i* and *j*. Then we have:

(12) $$W_{ij}^{1}=\{\begin{array}{c} 1/d_{ij}^{2},\;i\neq j\\ 0,\;i=j \end{array}$$

where $d_{ij}$ is the geographical distance between provinces, proxied by the distances of capital cities in each province.

### 4.2. Model Specifications

First, we examine the influence of financial development on carbon intensity without considering the mediating effect of technology. Transforming the log form of STIRPAT model into the linear form, we substitute the environment variable *I* in Equation (1) with carbon intensity variable *CI* and add other control variables to fit it into a spatial lag panel data model as Equation (13):

(13) $$CI_{it}=\alpha_{0}+\alpha_{1}FD_{it}+\sigma_{1}WCI_{-it}+\beta_{1}Open_{it}+\beta_{2}FDI_{it}+\beta_{3}Gov_{it}+\beta_{4}Reg_{it}+\lambda_{i}+\eta_{t}+\varepsilon_{ij}$$

where *W* is the geographic spatial weigh matrix; $WCI_{-it}$ is about the regions other than province *i* and specifically those who are neighbors; α and β are the coefficients for explanatory and control variables, respectively; σ is the spatial-autoregressive coefficient, which explains the clustering effect of carbon intensity between provinces. $\lambda_{i}$ and $\eta_{t}$ are spatial fixed effects, representing province- and year-fixed effects, respectively.

Next, following the causal stepwise regression approach proposed by Baron and Kenny [58], we construct the models as Equations (14) and (15) to examine the mediating effect of industrial upgrading and technological innovation.

(14) $$M_{it}=\beta_{0}+\beta_{1}FD_{it}+\sigma_{2}WM_{-it}+\underset{j}{\sum }\beta_{j}Control_{it}+\lambda_{i}+\eta_{t}+\varepsilon_{ij}$$

(15) $$CI_{it}=\gamma_{0}+\gamma_{1}FD_{it}+\gamma_{2}M_{it}+\sigma_{3}WCI_{-it}+\underset{j}{\sum }\gamma_{j}Control_{it}+\lambda_{i}+\eta_{t}+\varepsilon_{ij}$$

where *M* is the mediating variable (industrial upgrading or technological innovation); *Control* includes the variables defined in Equation (13).

## 5. Empirical Analysis

### 5.1. Spatial Autocorrelation Analysis

The statistical test results of Moran’s I and Geary’s C are shown in Table 3. Both values are significantly positive from 2006 to 2019, indicating that carbon intensity exhibits strong positive spatial autocorrelation between provinces in China. Therefore, one province’s carbon intensity is influenced by its neighboring provinces. The spatial distribution of emission intensity is not random but shows a clustering phenomenon in geographical space. The values of Moran’s I and Geary’s C show small fluctuations in each year but remain stable.

**Figure 5:**
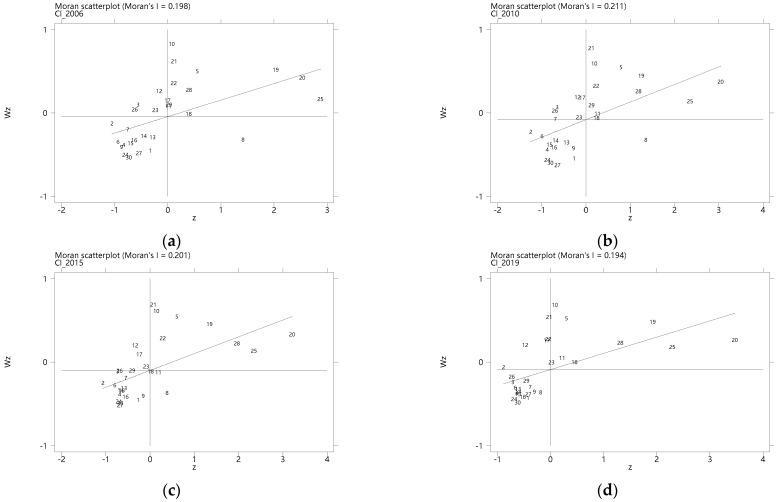
(**a**–**d**) Moran scatterplots of carbon intensity in 2006, 2010, 2015, 2019.

Furthermore, the Moran scatterplots of carbon intensity in 2006, 2010, 2015, and 2019 are drawn, respectively, based on the geographical spatial weight (The number of 1–30 in Moran scatterplot represents the 30 provinces in China, respectively: Anhui, Beijing, Chongqing, Fujian, Gansu, Guangdong, Guangxi, Guizhou, Hainan, Hebei, Heilongjiang, Henan, Hubei, Hunan, Jiangsu, Jiangxi, Jilin, Liaoning, Inner Mongolia, Ningxia, Qinghai, Shaanxi, Shanxi, Sichuan, Shandong, Shanghai, Tianjin, Xinjiang, Yunnan, Zhejiang.), as shown in Figure 5.

The first and third quadrants denote a positive spatial correlation, that is, provinces with high (low) carbon intensity are surrounded by neighboring provinces with the same attributes, which can be seen as the agglomeration areas of carbon emissions. The second and fourth quadrants represent a negative spatial correlation of high and low carbon emission provinces, that is, provinces with high (low) carbon intensity have low (high) carbon intensity in their neighboring provinces, which can be regarded as the dispersion area of carbon emission. Over the years, the quadrant and position of each province have not changed significantly, indicating that there is a relatively stable spatial dependence of provincial carbon intensity in China.

### 5.2. Spatial Lag Effect Analysis

#### 5.2.1. Full Sample Analysis

Table 4 reports the test results of the spatial lag model (SLM) for 30 provinces in China, and the heterogeneous estimations for sub-regions. Column (1) in Table 4 shows the results of SLM using the full sample dataset. The coefficient of *FD* is significantly negative at −0.2909, suggesting that for every 1% increase of the financial development level, carbon intensity will be reduced by about 0.29%.

W × CI has a significant positive influence on CI, with a coefficient of 0.4612, showing that for every 1% increase in the carbon intensity in other provinces, the carbon intensity of the local area will increase by about 0.46%. It reveals a positive spatial spillover effect of the carbon intensity among areas. Specifically, if in one province is higher, carbon intensity in the surrounding provinces is also higher, which is consistent with the spatial autocorrelation test results.

Of the control variables, the coefficients of Open, FDI, and Reg are not significant, indicating that China’s import and export, foreign investment contribution, and environmental regulation do not exhibit influence on China’s carbon emission reduction. The coefficient of Gov is significantly positive, indicating that government intervention has pushed up the carbon intensity in provinces to a certain extent.

#### 5.2.2. Sub-Regional Analysis

Columns (2)–(4) in Table 4 show the estimation results of eastern, central, and western regions in China, respectively (the eastern region includes Beijing, Fujian, Guangdong, Hainan, Hebei, Jiangsu, Liaoning, Shandong, Shanghai, Tianjin, Zhejiang. Central region includes Anhui, Henan, Heilongjiang, Hubei, Hunan, Jilin, Jiangxi, Shanxi. Western region in-cludes Gansu, Guangxi, Guizhou, Inner Mongolia, Ningxia, Qinghai, Shaanxi, Sichuan, Xinjiang, Yunnan, Chongqing.). The direction and significance of the coefficients of FD are consistent with the results of the full-sample estimation, indicating that the improvement of financial development contributes to the reduction of regional carbon intensity. However, the coefficient values of FD are slightly different across regions. Financial development in the central region has the greatest negative impact on carbon intensity, followed by the western and eastern regions.

The coefficients of W × CI in all sub-regions are consistent with the full sample analysis, that is, carbon intensity in neighboring provinces has a positive spillover effect on the local province. The estimated results of the control variables in eastern are not consistent with those in central and western regions: both import-export trade and environmental regulation in eastern region can significantly influence the intensity of emissions; but foreign investment and government intervention in eastern China have no significant impact on it. In the central region, the effect of government intervention is statistically positive. In the western region, both government intervention and environmental regulation intensify carbon emissions. These results imply that central and western regions in China may get trapped in a repetitive circle of “pollution-control-re-pollution”.

#### 5.2.3. Non-Linearity Analysis

Considering that there may be a non-linear relationship between financial development and carbon intensity, the quadratic term of financial development FD^2^ is introduced into the model as Equation (16):

(16) $$CI_{it}=\alpha_{0}+\alpha_{1}FD_{it}+\alpha_{2}FD_{it}^{2}+\sigma_{2}WCI_{-it}+\beta_{1}Open_{it}+\beta_{2}FDI_{it}+\beta_{3}Gov_{it}+\beta_{4}Reg_{it}+\lambda_{i}+\eta_{t}+\varepsilon_{ij}$$

**Figure 6:**
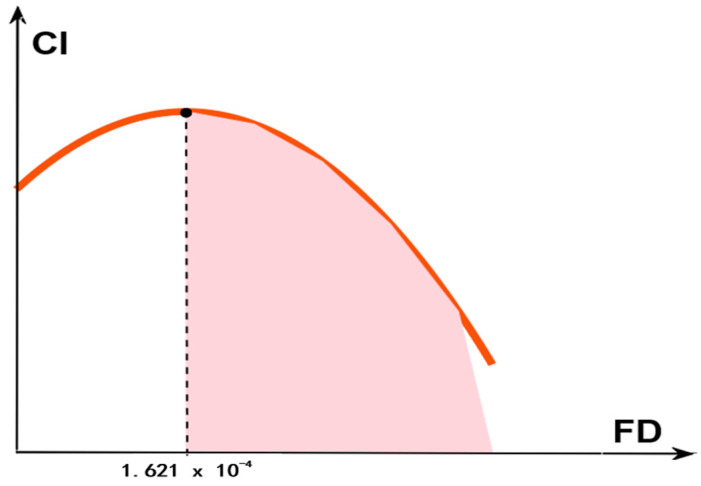
The inflection point of the inverted U-shaped relationship.

Table 5 reports the estimation results in non-linear spatial econometric models. The coefficient of FD in Column (1) is statistically negative. The coefficient of FD^2^ of the full sample is −0.0178, significantly negative at the level of 10%, indicating an inverted U-shaped relationship between financial development and carbon intensity. However, the inflection point is $e^{-\frac{-0.3107}{2\times \left(-0.0178\right)}}=1.621\times 10^{-4}$, as shown in Figure 6.

According to the indexes of FD, of the 30 provinces from 2006 to 2019, the minimum value of FD is 0.0424 at Qinghai in 2006, greater than the inflection point $1.621\times 10^{-4}$. It suggests the level of financial development at provinces in China completely lies in the right-half of the “inverted U”, therefore the impact of financial development on carbon intensity is still negative. The estimation result of the non-linear model is consistent with the linear model, indicating that the improvement of financial development in China is conducive to the reduction of carbon emissions intensity at the present stage.

Columns (2)–(4) in Table 4 show the sub-regional estimation results in the non-linear models. The coefficient value of FD^2^ in the eastern region is not significant, but statistically negative in the central and western regions. The overall results of the sub-regional analysis are still robust.

#### 5.2.4. Robustness Tests

Considering that different model settings may affect the estimation results of financial development on carbon emission intensity, this paper sets up three other types of spatial econometric models, which are spatial lag of X model (SLX), spatial error model (SEM), and spatial Durbin model (SDM). The test results of the above three models are shown in Columns (1)–(6) in Table 6. The impact of financial development on carbon emission intensity is still significantly negative, which further proves the robustness of the benchmark regression.

In addition, Beijing, Tianjin, Shanghai, and Chongqing have relatively concentrated financial resources, demographic dividend from a large population, strong economic vitality, and high achievements in economic development, which may cause the empirical results to be driven by the four municipalities. Therefore, to ensure the robustness of the findings, the samples of Beijing, Tianjin, Shanghai, and Chongqing are dropped out from the benchmark regression, and the results are shown in Columns (7) and (8) in Table 6, which again verifies the robustness of the results.

### 5.3. Mediating Effect Analysis

Table 7 shows the estimation results of the mediating effect models. Columns (1)–(4) and Columns (5) and (6) take industrial upgrading (Stru) and technological innovation (Tech) as the mediating variable, respectively.

The impact of financial development on industrial upgrading and technological innovation are reported in Columns (1), (3), (5), and (7). The coefficients of $\beta_{1}$, the effect of FD on Stru and Tech, are all significant and positive, suggesting that if the financial development level increases by an average of 1%, the degree of industrial upgrading will increase by 0.56–0.71% on average, and the regional innovative capability will increase by 0.76–0.8% on average. Therefore, financial development stimulates the rapid upgrading of regional industrial structure and the fast improvement of technological innovation. In addition, the upgrading of the industrial structure and technological innovation of the surrounding area also positively influence the local area. As can be seen, an average increase of 1% in the industrial upgrading and technological innovation of the neighboring provinces will lead to an increase of more than 0.4% and 0.5% in the local province and thus both industrial upgrading and technological innovation generate spillover effects.

The impact of financial development, industrial upgrading, and technological innovation on carbon intensity is reported in Columns (2), (4), (6), and (8). The coefficients of $\gamma_{1}$ and the effect of FD on CI are all significant and negative, suggesting that financial development reduces emission magnitude when considering the influence of industrial upgrading and technological innovation. As both $\beta_{1}$ and $\gamma_{1}$ are significant, a partial mediating effect occurs between financial development and carbon intensity. Therefore, the estimated results indicate that financial development can reduce carbon intensity partly through the upgrading of industrial structure and the improvement of technological innovation capacity.

## 6. Conclusions and Policy Implications

Based on the theoretical analysis of the impact of financial development on carbon intensity in China, we constructed the index of financial development, and employed a spatial lag model to estimate the spatial interaction between financial development and carbon intensity. Furthermore, we tested whether industrial upgrading and technological innovation mediate the relationship between the two. The conclusions are as follows. First, carbon intensity generates strong spatial spillover effects between provinces in China. Second, financial development in China significantly reduces carbon intensity. Even though the relationship presents an inverted-U relationship, as the level of provincial financial development in China currently lies in the right part of the “inverted U”, the overall effect of financial development on carbon intensity remains negative. In addition, sub-regional analysis shows the heterogeneity across regions: financial development has the greatest negative impact on carbon intensity in the central region, followed by the western and eastern regions. Third, the mediating effect test shows that financial development can reduce carbon intensity partly through industrial upgrading and technological innovation. Both of the mediating variables produce positive spatial spillover effects. All in all, financial development could effectively mitigate carbon intensity.

With the change of climate and the ongoing growth of China’s economy, China’s carbon dioxide emissions will continue to increase, but the intensity of carbon emissions is likely to decrease. China should make the best of financial development to alleviate carbon intensity and thus achieve the carbon peak goal. We put forward the following policy suggestions: first, inter-regional cooperation and communication on carbon emission reduction should be strengthened. As there exist big disparities across central, western and eastern China in financial development and carbon emission intensity, each region shall choose a proper direction and develop a leading industry based on its own conditions. The western and eastern regions may complement with the central region in both energy and financial development. Giving full attention to comparative advantages would jointly activate the synergistic effect of emission reduction. Second, financial institutions could actively develop environmentally friendly financial services and products, as well as broaden the funding channels and modes for emission reduction projects. Moreover, financial institutions could formulate green credit policies to help energy-intensive industries fulfill green transformation and development. Third, the government should coordinate industrial strategies and financial market policies to better promote the integration of industry, science technology, and finance.

The conclusions and policy implications in this article could provide a good reference as the world is seeking green and sustainable economy.

## Figures and Tables

**Table 1:** Entropy weighting of financial development indicators.

Index	Indicators	Entropy	Weight
Financial development	Deposits of the banking sector in RMB 100 million	0.934	0.074
	Loans of the banking sector in RMB 100 million	0.939	0.068
	Income of the insurance sector in RMB 100 million	0.933	0.075
	Expense of the insurance sector in RMB 100 million	0.934	0.075
	Proceeds of bonds in RMB 100 million	0.840	0.180
	Proceeds of shares in RMB 100 million	0.858	0.159
	Number of listed companies	0.922	0.088
	Number of financial institutions	0.970	0.034
	Fixed asset investment in RMB 100 million	0.912	0.099
	Average salaries of financial practitioners in RMB	0.982	0.021
	Number of financial practitioners in 10 thousand people	0.964	0.040
	GDP of the finance sector in RMB 100 million	0.922	0.087

**Table 2:** Variables and descriptive statistics.

Variable	Symbol	Meaning	Mean	SD	Min	Max
Carbon intensity	CI	ln (Carbon emission/real GDP)	0.9669	0.6504	−1.1597	2.5484
Financial development	FD	ln (Financial development index)	−0.4574	0.9882	−3.1616	1.8681
Industrial upgrading	Stru	ln (Industrial upgrading index)	1.8356	0.7516	−0.6687	3.4934
Technological innovation	Tech	ln (Technological innovation index)	−0.8907	1.4137	−4.5217	2.4249
Degree of openness	Open	ln (Imports and exports/GDP)	−1.7312	0.9723	−4.3662	0.5191
Foreign investment contribution	FDI	ln (FDI/GDP)	−4.2958	1.0880	−9.1150	−2.4892
Government intervention	Gov	ln (Fiscal expenditure/GDP)	−1.5731	0.4382	−4.3036	−0.4646
Environmental regulation	Reg	ln (Government regulation index)	−0.2166	0.8541	−3.9026	2.0702

**Table 3:** Spatial autocorrelation test results of carbon intensity between provinces in China.

Year	Moran’s I	Geary’s C	Year	Moran’s I	Geary’s C
2006	0.198 ***	0.778 ***	2013	0.218 ***	0.707 ***
2007	0.197 ***	0.793 ***	2014	0.208 ***	0.714 ***
2008	0.214 ***	0.768 ***	2015	0.201 ***	0.711 ***
2009	0.206 ***	0.763 ***	2016	0.208 ***	0.691 ***
2010	0.211 ***	0.747 ***	2017	0.200 ***	0.699 ***
2011	0.212 ***	0.729 ***	2018	0.212 ***	0.705 ***
2012	0.218 ***	0.714 ***	2019	0.194 ***	0.725 ***

Note: *** represent the significance level of 1%.

**Table 4:** Results of the impact of financial development on carbon intensity and the sub-regional heterogeneity analysis.

CI	Full Sample	Eastern Region	Central Region	Western Region
	(1)	(2)	(3)	(4)
FD	−0.2909 ***	−0.1100 ***	−0.2542 ***	−0.2557 ***
	(−12.27)	(−3.05)	(−6.25)	(−7.30)
W × CI	0.4612 ***	0.2490 ***	0.4193 ***	0.7178 ***
	(7.43)	(3.82)	(5.07)	(11.28)
Open	0.0221	0.6403 ***	−0.0615	0.0005
	(0.70)	(10.21)	(−1.23)	(0.01)
FDI	−0.0121	−0.0028	−0.0148	0.0146
	(−0.78)	(−0.14)	(−0.39)	(0.77)
Gov	0.1333 ***	0.0387	0.0752 **	0.5369 ***
	(3.64)	(0.41)	(2.47)	(6.39)
Reg	0.0019	−0.0900 ***	0.0249	0.0584 ***
	(0.15)	(−5.50)	(1.53)	(2.74)
Year-FE	Yes	Yes	Yes	Yes
Province-FE	Yes	Yes	Yes	Yes
Log-L	168.9820	105.7853	77.5682	89.8373
R^2^	0.4359	0.1832	0.2141	0.6007
N	420	154	112	152

Note: *** and ** represent the significance level of 1% and 5%, respectively. The z-statistics are in parentheses.

**Table 5:** Results of the impact of financial development on carbon intensity and the sub-regional heterogeneity analysis in non-linear models.

CI	Full Sample	Eastern Region	Central Region	Western Region
	(1)	(2)	(3)	(4)
FD	−0.3107 ***	−0.1054 ***	−0.3731 ***	−0.3103 ***
	(−11.84)	(−2.91)	(−7.30)	(−6.97)
FD^2^	−0.0178 *	−0.0127	−0.1055 ***	−0.0378 **
	(−1.78)	(−1.01)	(−3.76)	(−2.02)
W × CI	0.4402 ***	0.2586 ***	0.4001 ***	0.7086 ***
	(6.92)	(3.94)	(4.98)	(10.93)
Open	0.0218	0.6305 ***	−0.0224	0.0153
	(0.70)	(9.98)	(−0.46)	(0.46)
FDI	−0.0058	−0.0011	0.0207	0.0273
	(−0.37)	(−0.06)	(0.56)	(1.38)
Gov	0.1266 ***	0.0289	0.0560 *	0.4592 ***
	(3.45)	(0.31)	(1.93)	(5.02)
Reg	0.0025	−0.0903 ***	0.0313 **	0.0578 ***
	(0.20)	(−5.53)	(2.04)	(2.74)
Year-FE	Yes	Yes	Yes	Yes
Province-FE	Yes	Yes	Yes	Yes
Log-L	170.5592	106.2890	84.2467	91.8653
R^2^	0.4436	0.1744	0.3163	0.5186
N	420	154	112	152

Note: ***, **, and * represent the significance level of 1%, 5%, and 10%, respectively. The z-statistics are in parentheses.

**Table 6:** Results of robustness tests.

CI	SLX		SEM		SDM		Drop Four Municipalities	
	(1)	(2)	(3)	(4)	(5)	(6)	(7)	(8)
FD	−0.2250 ***	−0.2357 ***	−0.3662 ***	−0.3987 ***	−0.2152 ***	−0.2204 ***	−0.2705 ***	−0.3015 ***
	(-8.17)	(−7.80)	(−13.45)	(−14.41)	(−7.83)	(−7.37)	(−10.19)	(−10.11)
FD^2^		−0.0150		−0.0314 ***		−0.0142		−0.0223 **
		(−1.51)		(−2.98)		(−1.45)		(−2.34)
W × FD	−0.3433 ***	−0.2929 ***			−0.2598 ***	−0.1798 ***		
	(-8.57)	(−7.05)			(−5.16)	(−3.50)		
W × FD^2^		0.1206 ***				0.1359 ***		
		(4.54)				(5.16)		
W × Error			0.3104 ***	0.3041 ***				
			(2.88)	(2.88)				
W × CI					0.2180 ***	0.2864 ***	0.3893 ***	0.3497 ***
					(2.68)	(3.61)	(5.44)	(4.71)
Control-V	Yes	Yes	Yes	Yes	Yes	Yes	Yes	Yes
Year-FE	Yes	Yes	Yes	Yes	Yes	Yes	Yes	Yes
Province-FE	Yes	Yes	Yes	Yes	Yes	Yes	Yes	Yes
Log-L	178.7966	189.2574	149.2974	153.7013	182.2776	195.4101	190.4868	193.2105
R2	0.4049	0.4073	0.4173	0.4321	0.4203	0.4157	0.3969	0.4021
N	420	420	420	420	420	420	364	364

Note: *** and ** represent the significance level of 1% and 5%, respectively. The z-statistics are in parentheses.

**Table 7:** Results of the mediating effect test.

	Industrial Upgrading				Technological Innovation			
	(1)	(2)	(3)	(4)	(5)	(6)	(7)	(8)
	Stru	CI	Stru	CI	Tech	CI	Tech	CI
FD	0.5609 ***	−0.2831 ***	0.7113 ***	−0.3074 ***	0.8020 ***	−0.0607 *	0.7583 ***	−0.0814 **
	(8.82)	(−10.79)	(10.23)	(−10.22)	(20.49)	(−1.73)	(17.43)	(−2.24)
FD^2^			0.1383 ***	−0.0172 *			−0.0331 **	−0.0192 **
			(5.03)	(−1.65)			(−2.19)	(−2.06)
Stru		−0.0119		−0.0040				
		(−0.70)		(−0.23)				
W × Stru	0.4731 ***		0.4135 ***					
	(7.74)		(6.64)					
Tech						−0.2348 ***		−0.2362 ***
						(−8.37)		(−8.45)
W × Tech					0.5125 ***		0.5364 ***	
					(12.66)		(12.96)	
W × CI		0.4516 ***		0.4377 ***		0.2906 ***		0.2640 ***
		(7.07)		(6.77)		(4.48)		(3.98)
Control-V	Yes	Yes	Yes	Yes	Yes	Yes	Yes	Yes
Year-FE	Yes	Yes	Yes	Yes	Yes	Yes	Yes	Yes
Province-FE	Yes	Yes	Yes	Yes	Yes	Yes	Yes	Yes
Log-L	−233.4237	169.2240	−221.0764	170.5848	10.8907	201.7955	13.2670	203.9188
R^2^	0.3893	0.4397	0.4083	0.4446	0.8260	0.4959	0.8220	0.5028
N	420	420	420	420	420	420	420	420

Note: ***, **, and * represent the significance level of 1%, 5%, and 10%, respectively. The z-statistics are in parentheses.

## Data Availability

Data can be obtained from the corresponding authors upon request.
